# An Updated Review of Pro- and Anti-Inflammatory Properties of Plasma Lysophosphatidylcholines in the Vascular System

**DOI:** 10.3390/ijms21124501

**Published:** 2020-06-24

**Authors:** Eva Knuplez, Gunther Marsche

**Affiliations:** 1 Division of Pharmacology, Otto Loewi Research Center, Medical University of Graz, 8010 Graz, Austria; 2 BioTechMed-Graz, 8010 Graz, Austria

**Keywords:** lysophosphatidylcholine, inflammation, secreted phospholipases, biomarker, bioactive lipids

## Abstract

Lysophosphatidylcholines are a group of bioactive lipids heavily investigated in the context of inflammation and atherosclerosis development. While present in plasma during physiological conditions, their concentration can drastically increase in certain inflammatory states. Lysophosphatidylcholines are widely regarded as potent pro-inflammatory and deleterious mediators, but an increasing number of more recent studies show multiple beneficial properties under various pathological conditions. Many of the discrepancies in the published studies are due to the investigation of different species or mixtures of lysophatidylcholines and the use of supra-physiological concentrations in the absence of serum or other carrier proteins. Furthermore, interpretation of the results is complicated by the rapid metabolism of lysophosphatidylcholine (LPC) in cells and tissues to pro-inflammatory lysophosphatidic acid. Interestingly, most of the recent studies, in contrast to older studies, found lower LPC plasma levels associated with unfavorable disease outcomes. Being the most abundant lysophospholipid in plasma, it is of utmost importance to understand its physiological functions and shed light on the discordant literature connected to its research. LPCs should be recognized as important homeostatic mediators involved in all stages of vascular inflammation. In this review, we want to point out potential pro- and anti-inflammatory activities of lysophospholipids in the vascular system and highlight recent discoveries about the effect of lysophosphatidylcholines on immune cells at the endothelial vascular interface. We will also look at their potential clinical application as biomarkers.

## 1. Introduction

Formerly known as lysolecithins, elevated plasma levels of lysophosphatidylcholines (LPCs) were discovered in the 1950s in certain pathological conditions and were identified as a metabolic product of snake venom [1,2]. In contrast to phospholipids, LPCs are “cone-shaped”, with a polar “head” and a non-polar “tail” and therefore possess detergent-like properties [3]. The geometry of the LPC structure is also determined by the degree of saturation of the acyl chain. Combined, the saturation and length of the acyl chain is detrimental to its biophysical properties as well as its activity [4,5].

In addition to having non-specific membrane effects LPC was also reported to influence cell functions and activation status via binding to cell-specific G-coupled protein receptors (GPCRs) [6,7]. As of today, reports of LPC specifically binding to GPR119 [8], GPR40 (free fatty acid receptor 1) [9], GPR55 [9,10], GPR4 [11] as well as G2A [12] have been published. However, the study that reported binding of LPC to G2A [13] had to be withdrawn, because the authors could not demonstrate whether LPCs mediated its effects on cells directly via the receptor or via indirect membrane effects. Moreover, GPR4 was later found to be pH sensitive (proton-sensing GPCR) and results with LPC could not be reproduced [14,15,16]. More recent studies support the finding that binding of LPC or its derivates to GPR119, GPR40 and GPR55 induces intracellular calcium mobilization and leads to increased glucose-stimulated insulin secretion in different cell systems [9,17]. Interestingly, the authors demonstrated modulation of GPR40, GPR55 and GPR119 receptor binding affinities using phosphorothioate modified endogenous LPC [9]. When LPC was modified with a covalently bound *P*-anisic acid at the *sn-1* position, this increased its stability and decreased its toxicity showing the potential of LPC modification as a therapeutic option, when enhanced insulin secretion is needed [17].

Concentration of LPC in plasma and body fluids is already high under physiological conditions and reaches 100–300 µM [5,18]. LPCs are bound mainly to albumin and to a lesser extent to lipoproteins [19,20,21,22]. Inflammation, cell damage and other pathophysiological conditions can profoundly alter the ratio of free to albumin bound LPC through increased production of LPC or decreased plasma levels of albumin [23,24,25].

Plasma LPCs are bioactive lipid metabolites of phosphatidylcholine, which are mainly produced by the action of secretory phospholipases A2 (sPLA_2_) after removal of a fatty acid [26]. LPCs are also produced by the action of HDL-associated lecithin-cholesterol acyltransferase in the reverse cholesterol pathway [27], by the action of hepatic [28] and endothelial lipase [29] on lipoproteins as well as during lipoprotein oxidation [30]. The family of sPLA_2_ enzymes contains 10 catalytically active isoforms (IB, IIA, IIC, IID, IIE, IIF, III, V, X), which are differentially expressed in tissues and exhibit unique substrate selectivity. Of these, sPLA_2_-IIA is the only isoform detectable at higher concentrations in the bloodstream and is particularly elevated during inflammatory processes, triggering production of bioactive mediators of inflammation and resolution of inflammation [31,32]. One of the most well studied sPLA_2_ cleavage product beside LPC is arachidonic acid, which can be further converted via enzymatic (cyclooxygenase-I,-II and lipoxygenase) or non-enzymatic (auto-oxidation with reactive oxygen species) metabolism into prostaglandins, lipoxins and resolvins [33]. 

Interestingly, most of the recent studies, in contrast to older studies, found lower LPC plasma levels associated with unfavorable disease outcomes. Decreased levels of LPC were observed in rheumatoid arthritis [34], diabetes [35], schizophrenia [36], polycystic ovary syndrome [37,38], Alzheimer disease [39,40], pulmonary arterial hypertension [41], aging [42], asthma [43] and liver cirrhosis, where they were associated with increased mortality risk [44].

## 2. The Complex Role of LPC in Vascular Inflammation

### 2.1. Postulated Pro-Inflammatory Action of LPC on Vascular Reactivity

Endothelial cell dysfunction and subsequent changes in vascular reactivity are one of the earliest changes associated with atherosclerotic cardiovascular disease [45]. Oxidized low-density lipoprotein (ox-LDL) modified by the action of secretory phospholipase was found to inhibit endothelium-dependent relaxations [46]. Similar observations were made using free LPC, which was able to produce a defect in endothelium-dependent vasomotor regulation [47]. This could be explained by the finding that both ox-LDL [48] and LPC reduce the production of prostaglandin PGI_2_ in endothelial cells [49]. Subsequent research showed that reduced nitric oxide (NO) and not PGI_2_ production in endothelial cells is inhibited and responsible for the defects in vasorelaxation [50,51]. Others describe the involvement of proconstricting prostanoids and superoxide anions in LPC-attenuated vasorelaxation [52]. Not only endothelium-dependent vasorelaxation is reportedly impacted by LPC, but ox-LDL enriched in LPC can also independently cause vasoconstriction [53] or potentiate angiotensin II induced vasoconstriction [54]. It must be noted that ox-LDL consists of a complex mixture of many oxidized lipids and protein oxidation products in addition to LPC. This yields inconsistent results because, as Rao et al. showed [52], the potency as well as the underlying mechanisms of LPC-dependent attenuation of vasorelaxation is heavily dependent on the LPC acyl chain length and degree of saturation. 

### 2.2. Postulated Anti-Inflammatory Action of LPCs on Vascular Reactivity

In contrast to studies mentioned previously, reports of LPC inducing endothelium-dependent relaxation of smooth muscle cells via their non-specific membrane action have been published [55,56]. The induction of vasorelaxation was attributed to decreased endothelin-1 release, which acts as a potent vasoconstrictor [57]. Equally important was the finding that LPC induces cyclooxygenase-2 and endothelial nitric oxide synthase (eNOS) expression in endothelial cells, both of which can have vasoprotective effects either via production of prostacyclin or nitric oxide [58,59,60,61]. These observations indicate that LPC contributes to NO and endothelin-1 net balance, which regulates local vascular tone [62].

**Figure 1:**
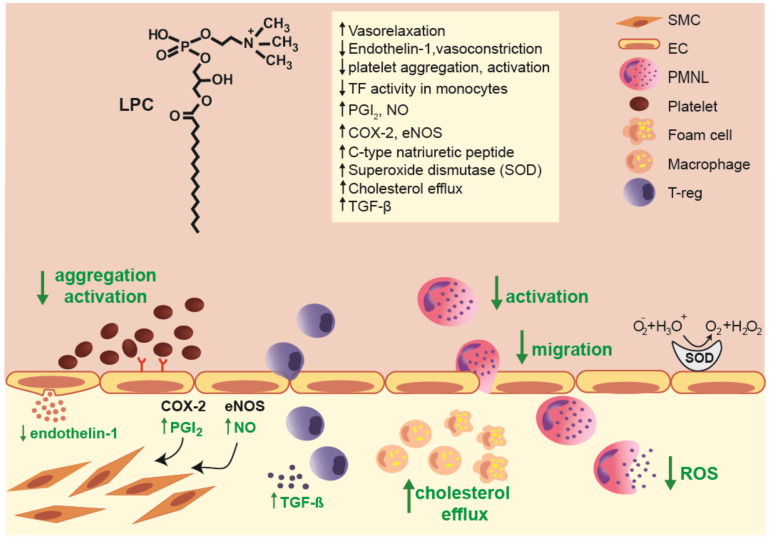
Overview of multiple anti-inflammatory effects of lysophosphatidylcholines (LPCs) at the vessel-endothelial interface. Abbreviations: COX-2, cyclooxygenase-2; EC, endothelial cell; eNOS, endothelial nitric oxide synthase; NO, nitric oxide; PMNL, polymorphonuclear leukocytes; ROS, reactive oxygen species; SMC, smooth muscle cells; TF, tissue factor; TGF-β, transforming growth factor beta; T-reg, T regulatory cells.

A recent study provided evidence that high-density lipoprotein (HDL) enriched with LPC (endothelial lipase modified HDL) increases eNOS activity by enriching the plasma membrane eNOS pool [63]. Moreover, LPC increased antioxidative capacity of HDL and protected LDL from oxidation [64]. In fact, contrarily to above mentioned older studies [48,49], in more recent studies LPC was shown to induce PGI_2_ production in endothelial cells [65]. These reports were confirmed in vivo, where it was shown that vascular relaxation induced by LPC administration was dependent on functional and morphological integrity of the vascular wall [66]. Moreover, LPC administration increased coronary blood flow as well as decreased mean arterial pressure and total vascular resistance in rabbit [67]. An overview of potential anti-inflammatory actions of LPC at the vessel-endothelial interface is shown in Figure 1.

### 2.3. Investigating the Effects of LPCs on Immune Cells Involved in Vascular Inflammation

Immune cells are involved in all stages of atherosclerosis and are a major contributor of atherosclerosis progression [68,69]. While immune cells are normally present in the vascular system, their quantity and activation status are increased in atherosclerotic lesions. Through their action and production of cytokines they are able to alter the endothelial inflammatory phenotype and contribute to structural instability of atherosclerotic plaques [68,70]. Older studies reported that LPCs may directly contribute to immune cell infiltration during vascular inflammation by increasing the expression of adhesion molecules such as intercellular adhesion molecule 1 (ICAM-1) [71,72], vascular cell adhesion protein 1 (VCAM-1) [73] or P-selectin [74], the expression of damage associated molecular patterns (DAMP) and MHC-II receptors [75] and by production of chemokines such as monocyte chemoattractant protein (MCP-1), IL-8, and Chemokine (C-C motif) ligand 5 (CCL5), also known as RANTES from endothelial cells. Furthermore, LPC was found to act as a strong chemoattractant for monocytes [76,77], T cells [78] as well as natural killer (NK) cells [79], attracting them to sites of inflammation. 

Similar to reports investigating the effects of LPC on vascular reactivity and endothelial activation, there are also contradictory reports on the effect of LPC on immune cells. 

#### 2.3.1. Effects of LPCs on Innate Immune Cells

Innate immune cells mainly consisting of monocytes and macrophages are the major players involved in the initiation and progression of vascular inflammation [80]. Studies performed on monocytes and macrophages on one hand show that LPC is able to activate macrophages, increase their phagocytic activity in the presence of T lymphocytes [81] and polarize them towards an M1-like phenotype [82]. Moreover, LPC was found to promote the release of arachidonic acid from monocytes [83] and regulate genes in the cholesterol synthesis pathway [84]. On the other hand, LPC was found to abrogate IL-6 release following lipopolysaccharide (LPS) stimulation [85] as well as to down-regulate platelet-activating factor (PAF) receptor expression and LPS-induced NF-κB translocation to the nucleus in monocytes [86]. A possible explanation for the seemingly confounding reports in monocytes was published by Carneiro et all where they proposed LPC serves as a dual-activity ligand molecule. LPC directly activates toll-like receptor (TLR) 4 and TLR-2-1 receptors in the absence of classical TLR ligands; however it inhibits TLR-mediated signaling in the presence of classical TLR ligands thereby acting as anti-inflammatory [87]. The surprising discovery of LPC mediated cholesterol efflux from cholesterol-laden macrophages—a well-known atheroprotective function of HDL—further suggested potent anti-atherogenic effects of LPC [88,89]. 

The granulocyte population of innate cells consisting of mast cells, neutrophils and eosinophils represents a smaller fraction of infiltrating immune cells during vascular inflammation [80]. However, their secreted granules contain factors capable of potentiating tissue damage and inflammation and proteases capable of modifying the surrounding extracellular matrix and locally deposited lipoproteins [90,91]. Moreover, it was shown that activated neutrophils and eosinophils form extracellular traps in the vessel wall, which are implicated in the clinical severity of the coronary lesion [90,92].

According to older studies performed in the 1980s, LPCs enhance the oxidative burst and reactive oxygen production in neutrophils [93]. However, the ability of LPC to prime neutrophils is heavily dependent on the length of the acyl chain of the molecule [93]. A more recent study performed by Lin et al. demonstrated that the most abundant LPC species in plasma (16:0, 18:0 and 18:1) in fact inhibit reactive oxygen production and activation of neutrophils. Furthermore, they demonstrated that the observed effect markedly varies on the solvent used to prepare LPC. Additionally, they demonstrated the anti-inflammatory effects of LPC in an ex vivo lung perfusion model where LPC prevented lung vascular injury mediated by neutrophils [94]. Curcic et al. likewise demonstrated that LPCs (16:0, 18:0 and 18:1) potently and rapidly inhibit neutrophil effector responses [95]. Similar results could be observed using eosinophils, where some studies found LPCs to increase cell migration [96] and adhesion [97]. Others, using physiological LPC-albumin complexes, discovered the ability of saturated LPCs to inhibit the activation and migration of isolated human eosinophils in vitro and in vivo [98]. LPCs were also found to either potentiate mast cell activation and secretion [99] or to inhibit histamine release, serving as a endogenous membrane stabilizers [100]. Altogether, the multiple LPC-induced pro- or anti-inflammatory activities on immune cells make it difficult to draw clear conclusions at the moment. However, it appears that addition of LPC as physiological LPC-albumin or LPC-HDL complexes, as performed in more recent studies, in general demonstrate anti-inflammatory properties. 

#### 2.3.2. The Proposed Roles of LPC on the Adaptive Immune System

Adaptive immunity is defined by the presence of lymphocytes, consisting of T-cells and immunoregulatory cytokines, majorly influencing inflammation in the vascular wall and atherosclerosis disease activity and progression [101]. The involvement of T cells in atherosclerosis is supported by the discoveries that approximately 10% of T lymphocytes isolated from atherosclerotic lesions recognize oxidized LDL in an MHC-II class restricted manner [102] and that early lesions isolated from apolipoprotein E deficient mice show evidence of clonal T-cell expansion [103]. LPC, in contrast to other lysophospholipids, was found to specifically potentiate the activation of T lymphocytes, while having no effect on resting cells [104]. ROS production and chemokine receptor expression in human Jurkat T cells were similarly significantly increased upon LPC addition [105,106]. Furthermore, LPC enhanced IFN-γ secretion and gene expression in CD4^+^ and CD8^+^ T cells as well as increased CD40L and CXCR4 expression in CD4^+^ T cells [107,108,109]. This enhanced activation of effector T cells, exhibited by increased OX40-Ligand (OX40L) and IFN-γ secretion by LPC may augment the inflammatory response in atherosclerotic lesions. 

On the other hand, LPC enhances the suppressive function of human naturally occurring regulatory T cells through TGF-β production [110]. Several studies have demonstrated the atheroprotective role of T regulatory cells in murine models of atherosclerosis [111,112]. Moreover, the anti-inflammatory effects of TGF-β are supported by human clinical data showing patients with advanced atherosclerosis have less active TGF-β [113] and in experimental models, where application of anti-TGF-β blocking antibodies accelerated the development of atherosclerotic lesions [114]. Altogether LPC involvement in T-cell mediated inflammation is yet unclear. From augmenting the activation of effector T cells in the early stages of inflammation to aiding in immunosuppression by T regulatory cell activation, LPC might serve as an endogenous homeostatic factor potentiating specific T-cell responses as needed.

### 2.4. Additional Anti-Inflammatory Effects of LPC in Vascular Inflammation and Atherosclerosis Development

When activated, platelets adhere to the endothelial monolayer and set a variety of inflammatory mediators, promote atherogenesis and increase vascular inflammation [115,116]. Reports of LPC possessing potent anti-aggregatory effects on platelets originate from the 1960s [117]. More recent studies confirm that modification of lipoproteins by secretory phospholipases inhibit platelet activation and aggregation and identify LPC as playing an essential role in the observed effects [118,119]. Furthermore, different LPC species were found to dose-dependently inhibit platelet aggregation induced by different agonists [119]. Coagulation is a complex process [120] and LPC was found not just to inhibit platelet aggregation, but also to reduce tissue factor activity in monocytes thereby attenuating coagulation in atherosclerotic lesions [121]. Additional vasoprotective and anti-inflammatory effects of LPC include increased expression of extracellular superoxide dismutase, which is important for antioxidant capacity of vascular walls [122] and of C-type natriuretic peptide, which inhibits the migration and proliferation of vascular smooth muscle cells [123]. Finally, the ability of LPC to bind C-reactive protein (CRP) and therefore suppress its pro-atherogenic effect on macrophages and delay the progression of atherosclerosis was reported [124]. A list of potentially beneficial effects of LPCs in relation to vascular inflammation and tumor formation is given in Table 1.

## 3. Future Directions of LPC as a Biomarker

In contrast to older studies, most of the more recent studies using mass spectrometry to quantify LPC subspecies mainly reported that decreased plasma LPC levels are associated with unfavorable disease outcomes. Decreased levels of LPC were observed in rheumatoid arthritis [34], diabetes [35], schizophrenia [36], polycystic ovary syndrome [37,38], Alzheimer disease [39,40], pulmonary arterial hypertension [41], aging [42], asthma [43] and liver cirrhosis, where they were associated with increased mortality risk [44]. Correspondingly serum metabolic profiling of patients undergoing treatment for schizophrenia discovered an increase in LPC following successful pharmacologic intervention [134]. Similarly, plasma LPC levels are decreased in sepsis [135] and correlate inversely with sepsis mortality [136] and in-hospital mortality in pneumonia [137]. Additionally, higher blood concentrations of LPC are positively correlated with the muscle insulin sensitivity index in diabetic patients [138] and inversely correlate with impaired fasting glucose and diabetes incidence [139,140,141,142]. Importantly, a reduction in LPCs was associated with a risk of adverse outcome in chronic kidney disease patients [143].

In the context of cancer research decreased levels of certain LPCs were identified as potential biomarkers in colorectal cancer [144,145,146], hepatocellular carcinoma [147], ovarian cancer [148,149], cholangiocarcinoma [150], pancreatic and biliary tract cancer [151] as well as cervical cancer [152]. Of particular interest, LPC levels proved to correctly predict the recurrence of prostate cancer after surgery [153]. LPC levels are decreased in cancer, associated with weight loss and increased inflammation, where they inversely correlate with CRP levels in plasma [22]. In lung cancer, decreased LPC levels were observed in malignant compared to benign pleural effusion [154]. Furthermore, a prospective metabolomics study discovered that higher levels of saturated LPC 18:0 reduced the risk of most common cancers [155], while higher levels of 8 different LPCs correlated with lower risk of advanced stage prostate cancer [156]. To enable high-throughput and quantitative analysis of LPCs as cancer biomarkers a novel parylene matrix biochip was recently developed and validated for clinical diagnosis [157].

## 4. Conclusions

Research into bioactive LPCs often resulted in contradicting data, even from experiments performed in the same disease model or cell type. Older studies that suggested that LPC could negatively affect many inflammatory diseases led research to look for treatments to lower LPC levels. Plasma levels of sPLA_2_-IIa correlate with cardiovascular risk and it is, therefore, thought to be involved in the pathogenesis of atherosclerosis [158,159,160,161,162]. This assumption suggests that a therapeutic intervention targeting sPLA_2_ could lead to favorable therapeutic effects for the patients. Indeed, a large clinical trial using the sPLA_2_ inhibitor varespladib (LY315920) targeting sPLA_2_ groups IIa, V and X did decrease lipid biomarkers as expected, which theoretically should have translated to a lower propensity to plaque rupture in the 16 weeks following acute coronary syndromes [163]. However, the study was terminated early "for futility" after an interim analysis of the outcomes for only 212 of the 5012 randomized patients [163]. In contrast, the use of varespladib was found to increase the probability of myocardial infarction, stroke and mortality in patients with acute coronary syndrome and acute coronary disease [163]. Moreover, clinical trials using the sPLA_2_ inhibitor varespladib found no evidence of beneficial effects for the treatment and prevention of sickle cell disease (NCT01522196), asthma [164], rheumatoid arthritis [165] and acute coronary syndrome [163]. The use of Varespladib was even associated with higher events of the composite primary outcome (cardiovascular mortality, nonfatal myocardial infarction, nonfatal stroke or unstable angina requiring hospitalization) and the trial was terminated for potential harm [163]. Hence, a more unbiased approach is needed to understand LPC in the context of homeostasis and disease pathology. 

Possible explanations for the discrepancy of data include use of different LPC species in regards to acyl chain length and saturation, which can impact their biological activity and function as shown by Frank et all in different models [52,59,65]. Since the free form of LPC is biologically most active [18], the results strongly depend on the presence and concentration of carrier proteins such as albumin or on the presence of lipoproteins in the experimental setup. Moreover, it was shown that a lot of reported pro-inflammatory effects attributed to LPC actually arise from PAF-like activity from contaminating phospholipids in some commercial preparations of LPC [166]. When these preparations were submitted to PAF acetylhydrolase or saponification (thereby targeting the susceptible *sn*-2 residue in PAF) the pro-inflammatory activity of the LPC preparations was abolished. 

Another important, but often overlooked issue in older studies, is the rapid conversion of LPC in biological fluids, tissue and living cells to phospholipids [167] or to lysophosphatidic acid (LPA) through the action of autotaxin [168]. In fact, LPC is the main substrate for the production of LPA, which is able to signal through G protein-coupled receptors and is implicated in chronic inflammation, fibrotic diseases and thrombosis [169,170]. Of particular interest in this context are recently published studies that link the pro-atherogenic effects of LPC to the effect of LPA on its receptors. It may well be that LPA and not LPC is responsible for the reported effects of LPC and ox-LDL on the development of atherosclerosis. [171,172,173,174,175]. Small molecule inhibitors of autotaxin, a secreted phosphodiesterase that produces LPA from LPC, and thus increasing LPC levels - are a new promising therapeutic option. Autotoxin inhibitors, which are currently entering phase III clinical trials for idiopathic pulmonary fibrosis have been extensively reviewed elsewhere [176]. Results of future studies employing autotaxin inhibitors are eagerly awaited.

When interpreting the available clinical and biomarker data and many newer experimental studies, it is clear that LPCs cannot be described simply as pro-inflammatory mediators, as anti-inflammatory activities often predominate. We therefore suggest that LPCs should instead be recognized as important homeostatic mediators involved in all stages of vascular inflammation through their effect on vascular reactivity, endothelial activation and infiltration, and activation of immune cells. Like with everything in nature it is impossible to paint a completely black and white picture of LPC due to the complexity of its interactions with a plethora of immune cells and its involvement in various processes. Nonetheless, the advancement of methods designed with appropriate controls as well as the use of stable LPC analogues has clearly aided in greater understanding of LPC actions in health and disease. 

## Figures and Tables

**Table 1:** Proposed favorable functions of lysophosphatidylcholines related to vascular inflammation and tumor development.

Function/Action	Tissue/Cell Type Studied	LPC Species Examined
Inhibition of platelet aggregation [118,119]	platelets	Mixture [118]; LPC 16:0, LPC 18:0, LPC 18:1, LPC 18:2 [119]
Decreased tissue factor activity and NF-κB expression [121]; Increased expression of extracellular superoxide dismutase [122]; Suppression of IL-6 release following lipopolysaccharide (LPS) stimulation [85]; Down-regulation of platelet activating factor (PAF) receptor expression and NF-κB translocation to nucleus [86]; Decreased high-mobility group protein 1 (HMGB-1) production [125]	monocytes	LPC 16:0 [121]; Mixture [122]; Not listed [85]; Mixture [86]; LPC 18:0 [125]
Increase in cholesterol efflux [88,89]	macrophage foam cells	LPC 14:0, LPC 16:0, LPC 18:0 [88]; Not listed [89]
Vascular smooth muscle relaxation [55,56]; Decrease in mean arterial pressure and coronary, renal and total vascular resistance [67]	rabbit aortic strip [55,56] in vivo application in rabbit [67]	Mixture [55]; LPC 10:0, LPC 14:0, LPC 16:0, LPC 18:0, LPC 18:1 [56]; Not listed [67]
Suppression of endothelin-1 secretion [57]; Increased prostacyclin production [58,65]; Increase in NO production [60,61]	endothelial cells	LPC 16:0 [57,58,60]; LPC 16:0, LPC 18:1, LPC 20:4 [65]; Not listed [61]
Increased C-type natriuretic peptide expression [123]	vascular smooth muscle cells	Mixture
Promotion of dendritic cell maturation [126]; Reduction of cell motility and adhesion [127]	dendritic cells	Mixture [126]; LPC 18:0 [127]
(Potentiated) T-cell activation [104,105,106,107,108,109]; Maintenance of T-cell homeostatic turnover [128]; Enhanced suppressive function [110]	T cells [104,109] CD4+ T cells [105,106,107,108] CD8+ T cells [128] regulatory T cells [110]	Mixture [104,105]; LPC 16:0 > LPC 18:0 > LPC 14:0 > LPC 18:1 [106]; LPC 16:0 [107,108,109]; LPC 11:0 [128]; Not listed [110]
Increased cytotoxic activity towards tumor cells [129]	NK cells	Not listed [129]
Inhibition of histamine release [100]	mast cells	LPC 16:0
Increased bactericidal activity [130], increased reactive oxygen species (ROS) production [93,131]; Decreased ROS production [94]; Inhibition of activation and effector functions [95]	neutrophils (PMNL)	Mixture [93]; LPC 18:0 [130]; Mixture, LPC 14:0, LPC 16:0, LPC 18:0 [131]; LPC 16:0, LPC 18:0, LPC 18:1 [94]; LPC 18:0 [95]
Inhibition of migration and effector functions [98]	eosinophils	LPC 16:0, LPC 18:0 [98]
Tumor cell apoptosis [132]; Reduction in tumor cell migration and adhesion [133]	tumor cells	Mixture [132]; LPC 16:0, LPC 18:0 [133]

## Abbreviations

eNOS	Endothelial Nitric Oxide Synthase
HDL	High-Density Lipoprotein
LPA	Lysophosphatidic Acid
LPC	lysophosphatidylcholine
Ox-LDL	Oxidized Low-Density Lipoprotein
sPLA_2_	Secreted Phospholipase A2
